# Community Built Environments and Cognitive Function: Evidence from a Nine-Year Longitudinal Study

**DOI:** 10.1093/geroni/igaf122.3228

**Published:** 2025-12-31

**Authors:** Jiaan Zhang

**Affiliations:** Fudan University, Shanghai, Shanghai, China (People’s Republic)

## Abstract

In recent years, increasing attention has been given to the role of community built environments in shaping health and well-being among older adults. This study examines the relationship between community built environments and cognitive function among Chinese older adults. Data were drawn from the China Health and Retirement Longitudinal Study (CHARLS) (2011–2020), a nationally representative sample of community-dwelling older adults. The analysis was restricted to individuals aged 60 and older at baseline, who were followed over a nine-year period. Cognitive function was assessed using a modified version of the Telephone Interview for Cognitive Status (mTICS), which evaluates episodic memory, numerical ability, orientation, and visuospatial skills. Community built environments were measured based on the availability of numbers of libraries, theaters, sports facilities, arts venues, and social organizations. Three-level mixed effects generalized linear models that account for clustering of observations within a subject over time and subjects within a community were employed for the study. The results showed that older adults residing in communities with greater availability of intellectually stimulating sites and sports facilities experienced a slower rate of cognitive decline over time, adjusting for individual-level demographic characteristics, socioeconomic status (SES), health characteristics and behaviors, and community-level SES ratings. This study highlights specific community infrastructure that may contribute to the preservation of cognitive function among older adults. The findings suggest the importance of incorporating cognitive health considerations into urban planning and community development initiatives in China.

